# Correction: Synthesis of non-toxic, biocompatible, and colloidal stable silver nanoparticle using egg-white protein as capping and reducing agents for sustainable antibacterial application

**DOI:** 10.1039/d0ra90124e

**Published:** 2020-11-26

**Authors:** Kalaiyarasan Thiyagarajan, Vijay K. Bharti, Shruti Tyagi, Pankaj K. Tyagi, Anami Ahuja, Krishna Kumar, Tilak Raj, Bhuvnesh Kumar

**Affiliations:** Defence Institute of High Altitude Research (DIHAR), Defence Research and Development Organization (DRDO) C/o 56 APO Leh-Ladakh-194101 India vijaykbharti@rediffmail.com +0172-2638900 +0172-2642900; Department of Biotechnology, Meerut Institute of Engineering & Technology Meerut Uttar Pradesh-250005 India; Defence Institute of Physiology and Allied Sciences, Defence Research and Development Organization (DRDO) Timarpur Delhi-110054 India

## Abstract

Correction for ‘Synthesis of non-toxic, biocompatible, and colloidal stable silver nanoparticle using egg-white protein as capping and reducing agents for sustainable antibacterial application’ by Kalaiyarasan Thiyagarajan *et al.*, *RSC Adv.*, 2018, **8**, 23213–23229, DOI: 10.1039/C8RA03649G.

The authors regret that an incorrect version of Fig. 3(D) was included in the original article. The correct version of Fig. 3(D) is presented below.

**Fig. 3:**
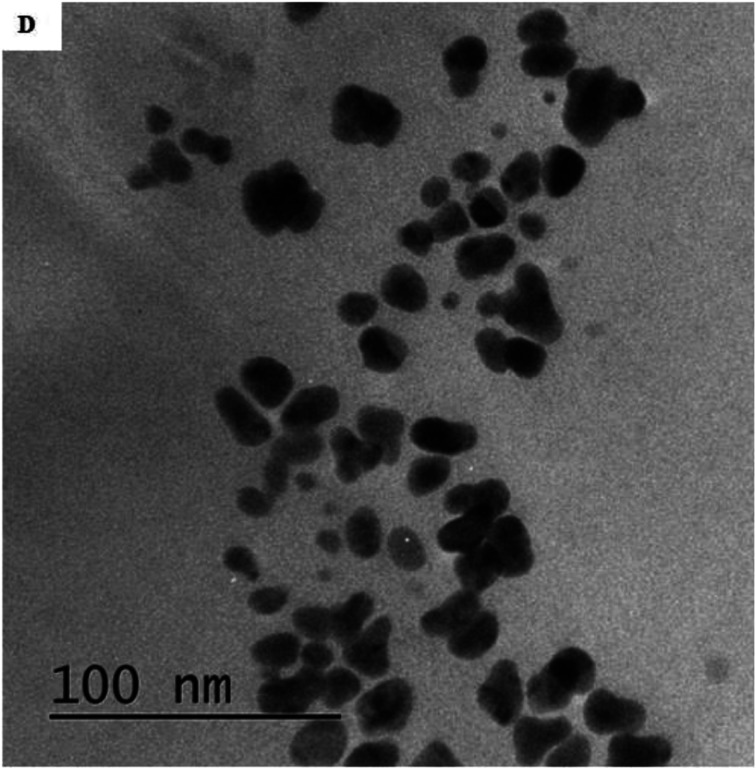
(D) TEM image of AgNPs-EW after freeze-drying.

The Royal Society of Chemistry apologises for these errors and any consequent inconvenience to authors and readers.

## Supplementary Material

